# Optimization of a reduced enzymatic reaction cascade for the production of L-alanine

**DOI:** 10.1038/s41598-019-48151-y

**Published:** 2019-08-13

**Authors:** Tobias J. Gmelch, Josef M. Sperl, Volker Sieber

**Affiliations:** 1 Chair of Chemistry of Biogenic Resources, Technical University of Munich, Campus Straubing for Biotechnology and Sustainability, Schulgasse 16, 94315 Straubing, Germany; 2 Catalysis Research Center, Technical University of Munich, Garching, Germany; 3 Fraunhofer Institute of Interfacial Biotechnology (IGB), Bio-, Electro- and Chemo Catalysis (BioCat) Branch, Straubing, Germany; 4 School of Chemistry and Molecular Biosciences, The University of Queensland, St. Lucia, QLD Australia

**Keywords:** Metabolic engineering, Metabolic engineering, Biocatalysis

## Abstract

Cell-free enzymatic reaction cascades combine the advantages of well-established *in vitro* biocatalysis with the power of multi-step *in vivo* pathways. The absence of a regulatory cell environment enables direct process control including methods for facile bottleneck identification and process optimization. Within this work, we developed a reduced, enzymatic reaction cascade for the direct production of L-alanine from D-glucose and ammonium sulfate. An efficient, activity based enzyme selection is demonstrated for the two branches of the cascade. The resulting redox neutral cascade is composed of a glucose dehydrogenase, two dihydroxyacid dehydratases, a keto-deoxy-aldolase, an aldehyde dehydrogenase and an L-alanine dehydrogenase. This artificial combination of purified biocatalysts eliminates the need for phosphorylation and only requires NAD as cofactor. We provide insight into in detail optimization of the process parameters applying a fluorescamine based L-alanine quantification assay. An optimized enzyme ratio and the necessary enzyme load were identified and together with the optimal concentrations of cofactor (NAD), ammonium and buffer yields of >95% for the main branch and of 8% for the side branch were achieved.

## Introduction

Sustainability is a major focus of modern day research, fighting a world governed by the demand on oil. To avoid a final depletion of fossil resources, renewable alternatives need to be identified and integrated into industrial production pathways. Promising directions are the bio-mimicking of natural pathways^1^ or the de novo design of artificial enzyme cascades^2^. They are considered powerful tools since natural evolution already found ways to convert a huge amount of substrates under nearly identical conditions^3,4^. Integrating multiple approaches for the conversion of biomass into biorefineries can further enhance their potential as a renewable platform for energy, fuels or chemicals^5,6^. Tailoring of enzymes towards specific problems additionally increases their versatility. In the last decades two inherently different process types evolved where either metabolically engineered whole cell approaches^7^ are applied or purified enzymes from various organisms are combined to *in vitro* enzyme reaction cascades. The latter approach has been termed “(*in vitro*) synthetic biosystems”^8,9^, “*in vitro* metabolic engineering”^10,11^ or “synthetic pathway design”^12,13^. Isolated biocatalysts are combined with the necessary cofactors in a controlled environment to produce valuable products^3,4,10^. With this approach the advantages of well-established *in vitro* biocatalysis are combined with the power of multi-step *in vivo* pathways to yield sophisticated biomanufacturing platforms^14^. These are less effected by cytotoxic compounds, do not have a membrane limited mass transport and can be directly controlled in contrast to classical microbial fermentations^15^. With the application of thermostable enzymes the time consuming protein purification can be reduced and the process stability at elevated temperatures is enhanced^9^. A wealth of successful examples that deploy cell-free enzymatic cascades for the production of e.g. alcohols^16^, chiral amines^17^, saccharides^18^ or hydrogen^12^ has thus been reported. The absence of a regulatory cell environment enables a direct process control and further optimization of such *in vitro* enzyme networks can dramatically improve the economy^8^. To identify the appropriate enzyme ratio and thereby avoid unnecessary expenses, one approach is a thorough kinetic analysis of the required biocatalysts including inhibition constants and pH effects as demonstrated by Beer *et al*.^19^. Next to this knowledge based strategy, Liu *et al*. described an empirical enzyme titration method, varying one enzyme at a time, in order to identify the best enzyme ratio for maximum productivity^20^.

**Figure 1:**
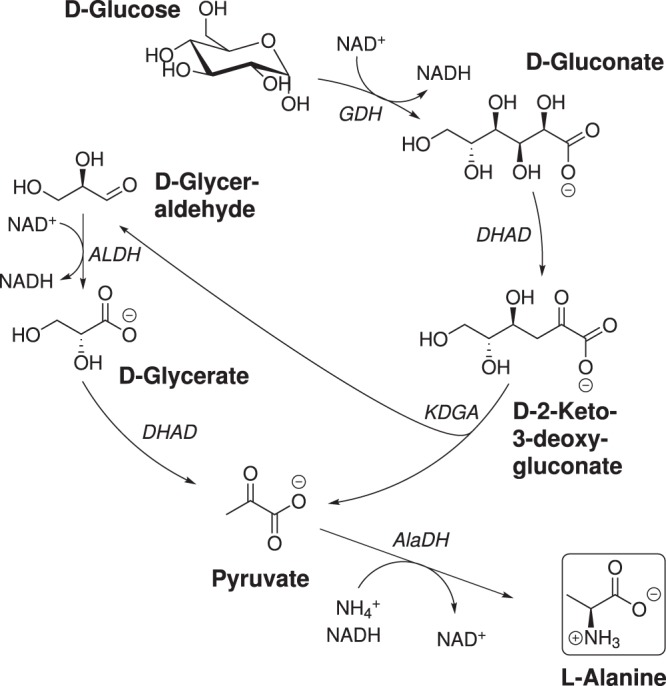
Schematic representation of the developed cell-free reaction cascade to L-alanine. Redox neutrality is achieved via combination of D-glucose and D-Glyceraldehyde oxidation with the reductive amination of pyruvate.

In this work, we designed a cell-free enzymatic cascade for the production of L-alanine directly from glucose and ammonium sulfate. As one of the smallest chiral compounds, L-alanine is used in various areas for example as food/ feed additive (L-alanine is the only L-amino acid with a sweet taste^21^), in health industry for nutrition therapies (e.g. main ingredient of Travasol^®^) and possibly as future feedstock for tailored thermoplastics^22,23^. Although various organisms have been shown to accumulate L-alanine^24^, the current industrial production relies on a resting cell biotransformation by *Pseudomonas dacunhae*^25^. κ-carragenan immobilized cells rendered this process quite feasible from an economic point of view, since the half-life of the derived biocatalyst is 260 d at 37 °C and an isolated L-alanine yield of >90% can be reached by a simple ion exchange resin purification step^26^. Nevertheless the substrate L-aspartic acid couples this process to the petroleum based production of ammonium fumarate^27^. In order to render this process sustainable, the design of our cell-free reaction cascade was inspired by the non-phosphorylative Entner-Doudoroff-Pathway derived from hyperthermophilic archaea^28^. We have previously described an artificial glycolytic cascade for the conversion of D-glucose to ethanol comprising only six enzymes without any need for phosphorylation and only NAD as redox shuttle^16^. Continuing this work we identified improved enzyme variants for the conversion of glucose via gluconate, 2-keto-3-deoxy-gluconate, glyceraldehyde and glycerate towards pyruvate. In combination with an L-alanine dehydrogenase from A*rchaeoglobus fulgidus* (EC 1.4.1.1, AfAlaDH) a redox neutral, L-alanine producing reaction cascade was obtained using only five to six enzymes (Fig. 1). For the final optimization of the enzyme ratio we combined the two approaches mentioned above and first determined the kinetic constants and then titrated the biocatalysts based on their maximum activity to achieve an overall increased efficiency.

## Results and Discussion

### Cascade design

A cell-free enzymatic reaction cascade for the conversion of D-glucose to L-alanine was developed from our previously published *in* vitro enzyme cascade to ethanol/ isobutanol^16^. In the first step, D-glucose is oxidized to D-gluconate under formation of NADH. D-gluconate then undergoes dehydration forming 2-keto 3-deoxy D-gluconate (KDG) followed by a retro aldol reaction resulting in one molecule pyruvate and one molecule D-glyceraldehyde. The aldehyde is oxidized producing another equivalent of NADH together with D-glycerate which is further converted to a second pyruvate molecule. While pyruvate was decarboxylated to acetaldehyde and further reduced to ethanol in the previously published cascade, we modified the last steps and directly integrated a reductive amination of pyruvate to yield L-alanine and recycle NAD simultaneously. With this redox neutral cascade two molecules of L-alanine can be formed from each molecule of glucose with NAD as the exclusive cofactor (Fig. 1). While the effects of ethanol or isobutanol on the enzymes can now be neglected, ammonium needs to be present for the formation of L-alanine and possible inhibitory effects on the biocatalysts need to be investigated. Inhibitory effects of L-alanine were not expected since most free amino acids tend to stabilize proteins^29,30^.

### Activity based enzyme selection

Learning from our ethanol cascade^16^ about the bottlenecks and drawbacks, our goal was to identify more effective enzyme variants for the pyruvate synthesis module. The combination of such an improved set of enzymes with a highly active L-alanine dehydrogenase should result in an optimized redox neutral, enzymatic cascade (Fig. 1).

At time point zero the cascade is started via the addition of D-glucose and NAD and the Glucose dehydrogenase (GDH) converts the initial substrates to D-gluconate and NADH. Previously, the GDH from the extremophile *Sulfolobus solfataricus* was applied because of its stability at the process temperature (50 °C). The v_max_ of SsGDH (15 U/mg) is in the lower range compared to other GDHs. Usually enzymes from mesophilic hosts show higher activity at moderate temperatures, but normally lack stability and degrade relatively fast. Bommarius *et* al. could increase the thermostability of a GDH from *Bacillus subtilis* by introducing two point mutations, while retaining its high specific activity^31^. Under the final process conditions this double mutant BsGDH was 14x more active towards D-glucose compared to our previously applied SsGDH, enabling a reduced enzyme load which resulted in an improved economy of the system. The following dehydration of D-gluconate towards KDG is catalyzed by a dihydroxyacid dehydratase (DHAD). In the ethanol cascade DHAD from *Sulfolobus solfataricus* was applied because of its activity for both relevant substrates D-gluconate and D-glycerate in combination with its high thermo-tolerance and excellent expression in *E*. *coli*. In another study we found that the preferred D-gluconate reaction is inhibited by D-glycerate, thus slowing down the entire cascade^32^. By applying two independent dehydratases, we tried to circumvent this problem and boost the overall cascade turnover. Since SsDHAD is to date the only biocatalyst that is able to catalyze the dehydration of D-glycerate, a substitution was not possible. We thus focused on the identification of an improved enzyme for the conversion of D-gluconate. Unfortunately we encountered solubility issues during the expression of archaeal gluconate dehydratases that are reportedly very active and stable against thermal degradation^33,34^. During our experiments a highly active mesophilic DHAD from *Caulobacter crescentus* (CcDHAD) was published^35^. In our hands CcDHAD was roughly 40x more active on D-gluconate than SsDHAD corresponding to a specific activity of 29 U/mg albeit at the cost of reduced thermostability. The product of the dehydration (KDG) is further subjected to a reversible retro aldol reaction. At this point the pathway is branched by an aldolase forming D-glyceraldehyde and pyruvate. Previously applied KDGA from *S*. *acidocaldarius* has a relatively high K_M_ for KDG (~14 mM) in combination with a moderate v_max_ (4 U/mg). From a range of candidate enzymes the highly specific aldolase from extremophile *P*. *torridus* (PtKDGA)^33^ with roughly 3 times higher activity was selected. With one pyruvate equivalent obtained from the KDG-splitting, one equivalent of NAD can directly be regenerated in the following reductive amination. For this last step a variety of L-alanine dehydrogenases is described in literature. Of these the enzymes from *Bacillus subtilis* and the extremophile *Archaeoglobus fulgidus* were selected for further investigations based on their described properties like activity and pH dependence^36,37^. While both biocatalysts can be produced in a soluble form in *E*. *coli* and have a pH optimum around pH 9, AfAlaDH was selected because of its excellent long term heat stability. Defined as the main branch this pathway via GDH, DHAD, KDGA and AlaDH can effectively run as a redox neutral cascade producing L-alanine and D-glyceraldehyde. Regarding the economy of our process, D-glyceraldehyde needs to undergo further conversion in order to eliminate the byproduct and double the theoretical L-alanine yield. The oxidation of the aldehyde was previously done by a mutated aldehyde dehydrogenase (ALDH) from *Thermoplasma acidophilum*^38^. With the original enzyme being strictly NADP dependent, a directed evolution mutagenesis resulted in some activity for NAD^39^. Although the K_M_ for NAD remained relatively high (~17 mM), the outstanding advantage of this enzyme for the ethanol cascade was its exclusive activity for D-glyceraldehyde with no side reactivity for the also occurring acetaldehyde. Since the alanine cascade does not depend on this exclusive activity anymore, we decided to use the ALDH of *Methanocaldococcus jannaschii* instead due to its higher expression yields and an improved specifity for NAD. The second step in the side branch is the dehydration of the D-glycerate to obtain another equivalent of pyruvate. While the natural glycolytic pathway requires phosphorylation we could previously demonstrate a successful deployment of SsDHAD for the direct dehydration of D-glycerate^16^. Although the activity is rather low (~10 mU/mg)^32^, this reaction enables our kinase-free strategy. Since D-glycerate is a strong competitive inhibitor for CcDHAD (K_I_ of ~0.5 mM), we tried to avoid its accumulation by applying the maximum possible amount of SsDHAD. The kinetic characteristics of all biocatalysts are summarized in Table 1.

**Table 1:** Michaelis Menten kinetics at 50 °C in 100 mM HEPES pH 7.35 with 100 mM ammonium sulfate; values in brackets represent previously published values for ethanol cascade enzymes^16^.

Enzyme	K_M_ (substrate) [mM]	K_M_ (cofactor) [mM]	V_max_ [U/mg]
BsGDH	7.9	0.5 (NAD)	218 (15)
CcDHAD	3.2 (7.8)	—	29 (0.7)
PtKDGA	1.1 (14)	—	12 (4)
MjALDH	0.3	0.3 (17)	2 (1)
AfAlaDH	0.2	0.06 (NADH) 113 ((NH_4_)_2_SO_4_)	26
SsDHAD^32^	7.8 (D-gluconate)	—	0.7 (D-gluconate) 0.01 (D-glycerate)

### Analysis of different ammonium sources

L-alanine formation was achieved via reductive amination of pyruvate with an L-alanine dehydrogenase from *Archaeoglobus fulgidus*. In our hands the K_M_ of AfAlaDH for ammonium was roughly 10x higher than reported in literature^36^. Because of that we had to identify a concentration of ammonium that promotes a high activity of AfAlaDH and simultaneously does not inhibit the other biocatalysts. In addition we realized different effects of the counterions on the activity of the biocatalysts of our cascade. To identify the ammonium source with least inhibition, activity assays were performed using ammonium chloride and ammonium nitrate as monovalent compounds and ammonium sulfate and diammonium phosphate respectively as bivalent ammonium sources. As shown in Table 2, BsGDH is generally activated by ammonium (+33% in case of ammonium chloride), while AfAlaDH is barely influenced by the type of ammonium source as long as ammonium is present. PtKDGA and CcDHAD show drastically reduced or no activity in presence of monovalent ammonium sources. Overall, ammonium sulfate is tolerated by all enzymes, retaining 60% and 70% activity for CcDHAD and PtKDGA. A final concentration of 200 mM ammonium was selected to be in the range of K_M_ for AlaDH while still not promoting further inhibition of the other enzymes.

**Table 2:** Activity of cascade enzymes depending on the ammonium source. Reactions were carried out in triplicates at 50 °C containing 100 mM HEPES pH 7.35 and either 5 mM NAD or 0.4 mM NADH and a final concentration of 200 mM ammonium (except H_2_O control). For BsGDH 100 mM glucose, for PtKDGA 1 mM KDG and an excess of MjALDH, for CcDHAD 20 mM gluconate and excess of PtKDGA/ MjALDH and for AfAlaDH 1 mM pyruvate were used. Average values are given in U/mL ± standard deviation.

[U/mL]	BsGDH	CcDHAD	PtKDGA	AfAlaDH
H_2_O	(4.4 ± 0.1)*10^3^	12.3 ± 1.6	41.6 ± 1.3	2.9 ± 1.5
NH_4_Cl	(5.8 ± 0.7)*10^3^	0	21.4 ± 2.8	91.0 ± 0.2
NH_4_NO_3_	(5.7 ± 0.1)*10^3^	0	4.8 ± 0.4	77.8 ± 1.0
(NH_4_)_2_HPO_4_	(5.0 ± 0.3)*10^3^	2.5 ± 0.4	31.5 ± 2.8	77.5 ± 3.1
(NH_4_)_2_SO_4_	(5.0 ± 0.1)*10^3^	8.3 ± 0.5	31.5 ± 0.2	86.2 ± 4.3

### L-alanine detection

The detection of amino acids (including L-alanine) in complex matrices is normally done via HPLC using precolumn derivatization with e.g. *o*-phtaldialdehyde (OPA)^40^. Since the run time per sample was relatively long, our aim was to identify a fast colorimetric assay, which is compatible with our enzyme and ammonium matrix. We compared a modified Berthelot reaction^41^, OPA, ninhydrin and fluorescamine in order to obtain the best product monitoring platform for our system. The Berthelot reaction refers to the alkaline derivatization of ammonium by phenol and hypochlorite in order to quantify free ammonium. From the residual ammonium concentration, we wanted to infer the alanine concentration but these measurements were unsatisfying. The other three compounds represent common reagents for the derivatization and quantification of amines. Especially primary amino groups can react with these detection agents, producing chromo- or fluorophores. Unfortunately, ammonia also reacts with these compounds and forms similar products, which interfere the alanine detection. For OPA and ninhydrin the excess of ammonium in the matrix had a significant influence on the measurement and prevented an accurate quantification of L-alanine. In contrast, fluorescamine^42^ derivatization showed sufficient selectivity for L-alanine over ammonium and was therefore selected to directly quantify the produced L-alanine without any prepurification (Fig. S1). Calibration was linear in the range of 1 to 30 mM L-alanine in complex enzyme/ ammonium matrix (Fig. S1). Further avoiding NADH fluorescence influence (λ_ex_ = 366 nm (in living culture), λ_em_ = 460 nm^43^), only endpoint measurements after 12 h have been taken.

### Initial L-Alanine production

Since we knew from the ethanol cascade that the D-glycerate dehydration is relatively slow, we decided to start the optimization of the cascade with the main branch including the enzymes BsGDH, CcDHAD, PtKDGA and AfAlaDH while D-glyceraldehyde was accumulated. A separate optimization should avoid the risk of an NAD sink, where every consumed NAD molecule is temporarily trapped until pyruvate is formed from glycerate.

**Figure 2:**
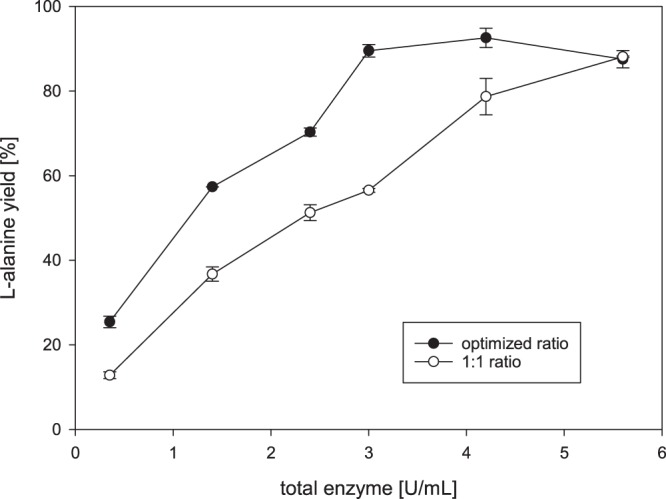
Comparison of L-alanine yields of different enzyme loads of the optimized enzyme ratio and a 1:1 enzyme ratio. The optimized ratio shows improved performance and higher L-alanine yields at identical enzyme load. Reaction contained either the optimized enzyme ratio BsGDH:CcDHAD:PtKDGA:AfAlaDH = 2:10:1:2 or a 1:1 ratio of all enzymes.

The starting conditions of 50 °C, 100 mM HEPES pH 7.35, 25 mM D-glucose and 5 mM NAD have been adapted from our previous cascade setup^16^. Together with 100 mM ammonium sulfate the enzyme reaction was incubated at 50 °C for 12 h. For the initial L-alanine production the activity of the biocatalysts was adjusted to be in a 1:1 ratio to each other and the sum of these activities was defined as total units within the cascade. Stepwise increase of the total enzyme load results in a linear increase of the L-alanine yield up to about 90% for 5.6 U/ml total enzyme (Fig. 2).

### Optimization of L-alanine production

**Figure 3:**
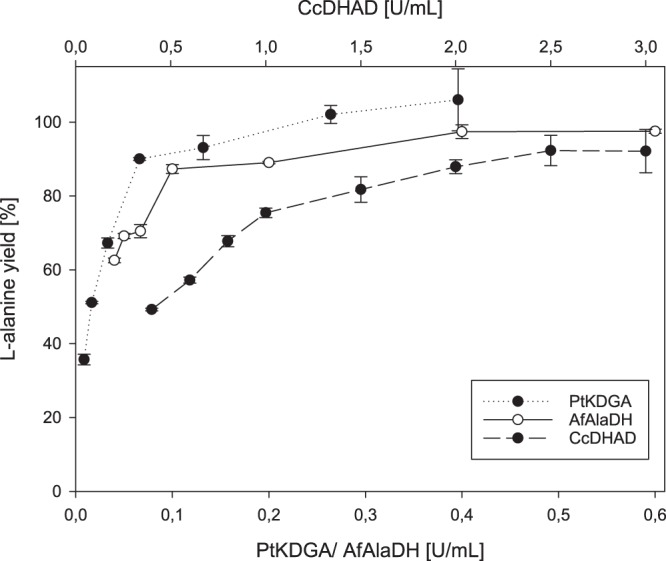
Single enzyme titration of CcDHAD, PtKDGA and AfAlaDH. The curves show the dependence of the L-alanine yield on the amount of single cascade enzymes.

**Figure 4:**
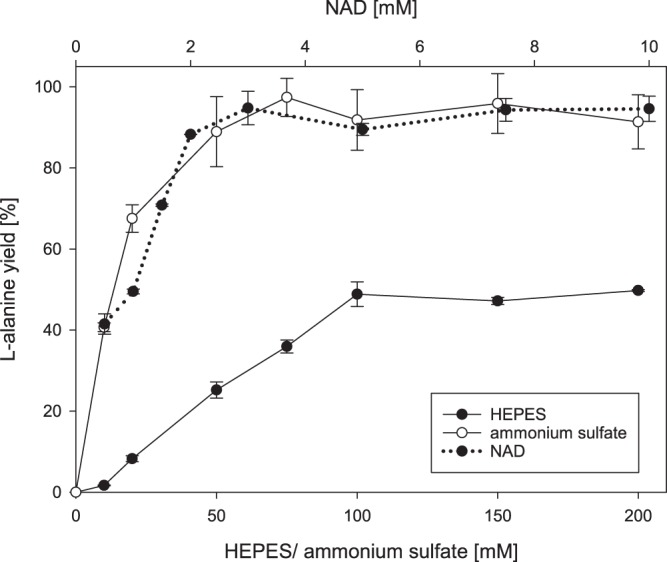
Dependence of the L-alanine yield on the concentrations of HEPES buffer, ammonium sulfate and NAD.

To improve L-alanine production from D-glucose while using a minimal amount of enzymes, an optimization of the enzyme ratio was performed followed by optimizations of cofactor, buffer and ammonium concentration. Applying the above mentioned starting conditions, the concentration of each enzyme was set to 0.4 U/mL and CcDHAD was increased incrementally. As depicted in Fig. 3, L-alanine yields increased steadily up to 90% with no further increase for volumetric activities higher 2 U/mL. Hence this enzyme concentration was used for the further optimization reactions. A similar optimization of PtKDGA resulted in roughly quantitative yields at 0.2 U/mL and AfAlaDH was ideal at 0.4 U/mL. This led to an optimized enzyme ratio of BsGDH: CcDHAD: PtKDGA: AfAlaDH = 2/10/1/2. Interestingly, the cofactor recycling enzymes BsGDH and AfAlaDH are present in a 1:1 ratio while only half of an equivalent PtKDGA is necessary. The required excess of CcDHAD is possibly due to its lower thermostability. In the next step we used the optimized enzyme ratio to determine the buffer, NAD and ammonium optima. The maximum L-alanine yield (97%) was reached for 3 mM NAD and 75 mM ammonium sulfate (Fig. 4). For the HEPES buffer optimization 50% of the above mentioned enzyme load were applied, to temporarily increase the buffer influence on the system. The results correlate to our other experiments and we found a linear increase of product yield until 100 mM, which is corresponding to a 4 fold excess compared to the 25 mM glucose load. This excess is necessary due to acid formation during the glucose oxidation pathway. Finally we repeated the total enzyme load experiment comparing the optimized to the 1:1 enzyme ratio (Fig. 2). Although both curves increase linearily up to above 90% of L-alanine yield, the optimized enzyme ratio reaches its maximum already at 3 U/mL, while almost the double amount of enzyme (5.6 U/mL) is necessary for the classic 1:1 ratio to obtain the same L-alanine yield. Time dependent experiments revealed that the production of L-alanine ceased after 6-8 h indicating enzyme inactivation. A possible explanation could be the modification of enzymes by the accumulated D-glyceraldehyde.

To avoid this problem we included MjALDH and SsDHAD into our set up trying to redirect the D-glyceraldehyde towards pyruvate. With these additional two enzymes the theoretical yield doubles to 50 mM L-alanine from 25 mM D-glucose. Due to the very poor activity of SsDHAD for D-glycerate, we decided to use the maximum possible amount of the enzyme resulting in 4 mU/mL final activity. Applying the optimized conditions from above, we stepwise increased the concentration of MjALDH. Interestingly a very low ALDH concentration of 25 mU/mL promotes the formation of 29 mM L-alanine, clearly indicating some activity in the side branch of the cascade. Increasing the concentration of MjALDH up to 0.2 U/mL resulted in a steady decrease of the L-alanine yield reaching a plateau around 12 mM. Since almost quantitative yields (24 mM L-alanine) could be demonstrated in the main branch, this additional pathway seems to have a negative impact on the entire cascade. With increasing ALDH concentrations, the D-glycerate and NADH production speeds up and an inhibition of CcDHAD by accumulated D-glycerate could explain the influence on the main branch (for experimental analysis of CcDHAD inhibition see Figure S2). Furthermore the regeneration of the now accumulated NADH is also limited to the D-glycerate dehydration since the *in situ* cofactor recycling via AlaDH does require Pyruvate. This leads to a steady decrease of the NAD pool, finally slowing down the entire cascade to the speed of the D-glycerate dehydration. A twostep process, where the main branch was run for 12 h before MjALDH and SsDHAD were added did not produce any additional L-alanine.

Nevertheless we could establish a redox neutral, *in vitro* enzymatic reaction cascade from D-glucose to L-alanine comprising six enzymes and NAD as the only cofactor. After identification and characterization of suitable biocatalysts, a thorough optimization of the enzyme ratio, total enzyme load, buffer-, NAD- and ammonium concentration doubled the cascade’s effectiveness resulting in a more cost efficient enzyme loading. By splitting the cascade into two branches, we obtained nearly quantitative yields (>95%) in the main branch via BsGDH, CcDHAD, PtKDGA and AfAlaDH plus additional 8% in the less effective side branch via MjALDH and SsDHAD and again AfAlaDH. During the reaction time of 12 h we obtained a space-time-yield of 0.17 g/(Lh) with a catalyst loading of 0.05 g of catalyst per g of L-alanine. This space-time-yield is still far behind current industrial alanine production from fumarate, where 13.4 g/(Lh) can be reached with immobilized cells from *E*. *coli* and *P*.*dacunhae*^44^. Engineered *E*. *coli* can form up to 121 g/L L-alanine during a 39 h fermentation resulting in a space time yield of 3.1 g/(Lh)^45^. Although we could demonstrate a successful redirection of the accumulated D-glyceraldehyde towards pyruvate, the dehydration of D-glycerate is still considered as the main bottleneck of this reaction cascade. In order to further increase the efficiency of the cascade and enable preparative scale production of L-alanine, identification of improved enzymes as well as enzyme engineering will be necessary.

## Materials and Methods

### Chemicals

D-glucose (monohydrate), sodium pyruvate, NAD and NADH (disodium salt) were obtained from Carl Roth GmbH and ammonium sulfate was purchased from Applichem. Sodium-D-gluconate and L-alanine were purchased from Sigma-Aldrich and fluorescamine was bought from Alfa Aesar. All chemicals were used as received and 2-keto-3-deoxy D-gluconate (KDG) was synthesized using 200 mM D-gluconate and the dihydroxyacid dehydratase from S*ulfolobus solfataricus* (SsDHAD) as described previously^32^.

### Strains and plasmids

*E*. *coli* BL21(DE3)(F^-^ ompT hsdSB (rB^-^ mB^-^) gal dcm (DE3)) cells were purchased from Novagen and used for protein production. For cloning *E*. *coli* DH5α (fhuA2 lac(del)U169 phoA glnV44 Φ80’ lacZ(del)M15 gyrA96 recA1 relA1 endA1 thi-1 hsdR17) was obtained from Invitrogen. The genes encoding for Dihydroxyacid dehydratase from *Caulobacter crescentus* (CcDHAD), 2-keto 3-deoxygluconate aldolase from *Picrophilus torridus* (PtKDGA), aldehyde dehydrogenase from *Methanocaldococcus jannaschii* (MjALDH) and alanine dehydrogenase from A*rchaeoglobus fulgidus* (AfAlaDH) were obtained codon optimized for *E*. *coli* from GeneArt Thermo Scientific and cloned into pET vectors via NdeI/ XhoI restriction sites or NcoI/ XhoI for MjALDH (Table 3). The plasmids coding for D-glucose dehydrogenase from *Bacillus subtilis* (BsGDH E170 K/Q252L)^31^ and SsDHAD^32^ have been prepared previously in our lab. All sequences can be found in the supplement.

**Table 3:** Overview of the applied enzymes and plasmids.

NO.	Plasmid	Enzyme	Abbrev.	Source
**1**	pACYC-Duet-BsGDH^31^	Glucose dehydrogenase E170K/Q252L	BsGDH	*Bacillus subtilis*
**2**	pET28a-CcDHAD	Dihydroxyacid dehydratase	CcDHAD	*Caulobacter crescentus*
**3**	pET28a-PtKDGA	2-keto-3-deoxygluconate aldolase	PtKDGA	*Picrophilus torridus*
**4**	pET24a-AfAlaDH	Alanine dehydrogenase	AfALaDH	*Archaeoglobus fulgidus*
**5**	pET28a-MjALDH	Aldehyde dehydrogenase	MjALDH	*Methanocaldococcus jannaschii*
**6**	pCBR-SsDHAD^32^	Dihydroxyacid dehydratase	SsDHAD	*Sulfolobus solfataricus*

### Enzyme expression

Enzyme expression was performed in *E*. *coli* BL21(DE3) as host strain in shaking flask cultures using ZYP-5052 autoinduction media^46^, supplemented with 100 µg/mL kanamycin or 10 µg/mL chloramphenicol. After 1%-inoculation of the autoinduction media with an overnight grown culture of *E*. *coli* BL21(DE3) harboring the corresponding plasmid, expressions were carried out over night at 30 °C and 120 rpm.

### Enzyme purification

Unless stated otherwise, all steps were carried out at room temperature (25 °C), since some proteins showed sensitivity towards lower temperatures. Protein concentration was measured by a Bradford protein assay using the Roti-Nanoquant reagent (Carl Roth GmbH) according to the manufacturer’s recommendations with bovine serum albumin as standard.

Cells were harvested, resuspended in 100 mM HEPES buffer pH 7.35 and disrupted by ultrasonication in an ice cooled water bath. Lysates of BsGDH, AfAlaDH and MjALDH were heat treated at 70 °C for 15 min, lysates of PtKDGA at 65 °C for 30 min. After clarifying the lysates via centrifugation, CcDHAD and PtKDGA samples were loaded onto a His GraviTrap column (GE Healthcare) equilibrated with 100 mM HEPES pH 7.35 containing 10 mM imidazole following the manufacturer’s recommendations. The elution buffer contained 500 mM imidazole and subsequently the eluted protein and the heat treated lysates were desalted using a PD-10 column (GE Healthcare) equilibrated with 100 mM HEPES pH 7.35. Purified proteins were flash frozen in liquid nitrogen, stored at -80 °C and thawed freshly every day. 17.1 µmol MnCl_2_ was added per mg of purified CcDHAD before freezing. SsDHAD purification was performed as described previously^32^.

### Activity assays

If not stated otherwise, all experiments contained 100 mM HEPES buffer pH 7.35, 100 mM ammonium sulfate pH 7 (both titrated with NaOH) and were preheated for 10 min at 50 °C before the reaction was started with the addition of the appropriate amount of enzyme diluted in 100 mM HEPES pH 7.35 containing 0.2 mg/mL BSA.

All enzymes were analyzed photometrically following NADH consumption or production at 340 nm (ε_NADH = _6.22 mM^−1^cm^−1^) with an Epoch 2 Microplate Spectrophotometer (BioTek GmbH) at 50 °C. If not mentioned otherwise, reactions were carried out in triplicates using 96 well plates (Greiner f-bottom) containing a final volume of 200 µL. Michaelis-Menten constants were determined for all enzymes by measuring eight different substrate concentrations, plotting initial velocities vs. substrate concentration and fitting the parameters with the online tool *ic50*.*tk*. One unit (1 U) is defined as the amount of enzyme that consumes 1 µmol of substrate per minute.

Kinetic measurements of BsGDH contained either 0.06–2.5 mM NAD (100 mM D-glucose) or 1–100 mM D-glucose (3 mM NAD). Reaction mixtures for determining the dependence of PtKDGA activity on the concentrations of 2-keto 3-deoxy D-gluconate (KDG) contained 1 mM NAD and KDG concentrations ranging between 0.03 and 3 mM together with sufficient amounts of purified MjALDH.

Kinetic parameters of AfAlaDH were determined in cuvettes (0.9 mL final volume) with reaction mixtures containing either 0.005-0.3 mM NADH (1 mM pyruvate, 100 mM (NH_4_)_2_SO_4_) or 0.1–1 mM pyruvate (0.2 mM NADH, 100 mM (NH_4_)_2_SO_4_) or 15–175 mM (NH_4_)_2_SO_4_ (1 mM pyruvate, 0.2 mM NADH).

Reaction mixtures for the determination of kinetic parameters of CcDHAD contained 0.2–20 mM D-gluconate, 1 mM NAD and an excess of purified PtKDGA and MjALDH.

### Analysis of different ammonium sources

To identify the most suitable ammonium source, reactions were carried out as described above (at maximum substrate concentrations) with a final concentration of 200 mM ammonium. 1 M stock solutions of NH_4_Cl, NH_4_NO_3_, (NH_4_)_2_SO_4_ and (NH_4_)_2_HPO_4_ were titrated with NaOH to a pH of 7 (at 25 °C) prior to use.

### L-alanine detection

Alanine assays were performed in a 96 f-bottom well plate (NUNC black) in a Varioscan plate reader (Thermo Fisher) according to a modified protocol from Bantan-Polak *et al*.^42^. 10 µL of diluted sample were mixed with 45 µL of a 0.1% fluorescamine solution in dry acetonitrile. After the addition of 8 µL 100 mM sodium borate buffer pH 10 and 95 µL of water, the fluorescence was measured at λ_em_ = 486 nm (λ_ex_ = 396 nm). Signal calibration was done using L-alanine concentrations ranging from 1-30 mM and the complex matrix of the reactions.

### Optimization of L-alanine production

One pot L-alanine synthesis reactions were set up in PCR-tubes containing 25 µL final volume and incubated in a C1000 Thermo Cycler (BIORAD) at 50 °C for 12 h. The initial reaction setup contained 25 mM D-glucose, 5 mM NAD, 100 mM HEPES pH 7.35, 100 mM ammonium sulfate pH 7 and 0.4 U/mL of each enzyme (BsGDH, CcDHAD, PtKDGA and AfAlaDH). BsGDH was kept constant while the activity of the other enzymes was varied. The tested enzyme concentration for the optimization was CcDHAD 0.4–3 U/mL, PtKDGA 0.013-0.8 U/mL and AfAlaDH 0.04-0.8 U/mL using the optimized values for following optimizations. In addition the total enzyme load (0.35-5.6 U/mL), the NAD concentration (0.5-10 mM), the ammonium sulfate concentration (0–200 mM) and the HEPES buffer concentration (0-200 mM) were optimized accordingly.

Reactions for investigating the full cascade contained the optimized conditions for the main branch (0.4 U/mL BsGDH, 2 U/mL CcDHAD, 0.2 U/mL PtKDGA, 0.4 U/mL AfAlaDH, 5 mM NAD, 100 mM HEPES buffer pH 7.35, 100 mM ammonium sulfate and 25 mM D-glucose). MjALDH was used at levels between 13-800 mU/mL in combination with 0.42 mg/mL SsDHAD.

## Supplementary information

Supplementary Information

## Data Availability

All data generated or analyzed during this study are included in this published article (and its Supplementary Information files).
