# Supplementary material for: Optimization of a reduced enzymatic reaction cascade for the production of L-alanine
Source: Sci Rep. 2019 Aug 13;9:11754. doi: 10.1038/s41598-019-48151-y (PMC6692406; doi:10.1038/s41598-019-48151-y)
Supplement: Supplementary file 1 — Supplementary Information [file 41598_2019_48151_MOESM1_ESM.pdf]

Supplementary Material

Optimization of a reduced enzymatic reaction cascade for the production of L-alanine

Tobias J. Gmelch[1], Josef M. Sperl[1], Volker Sieber[1,2,3,4]*

[1] Chair of Chemistry of Biogenic Resources, Technical University of Munich, Campus Straubing for Biotechnology and Sustainability, Schulgasse 16, 94315 Straubing, Germany
[2] Catalysis Research Center, Technical University of Munich, Garching, Germany
[3] Fraunhofer Institute of Interfacial Biotechnology (IGB), Bio-, Electro- and Chemo Catalysis (BioCat) Branch, Straubing, Germany
[4] School of Chemistry and Molecular Biosciences, The University of Queensland, St. Lucia, QLD, Australia

* Correspondence should be addressed to
Prof. Volker Sieber
E-Mail: sieber@tum.de
Tel: +499421187301

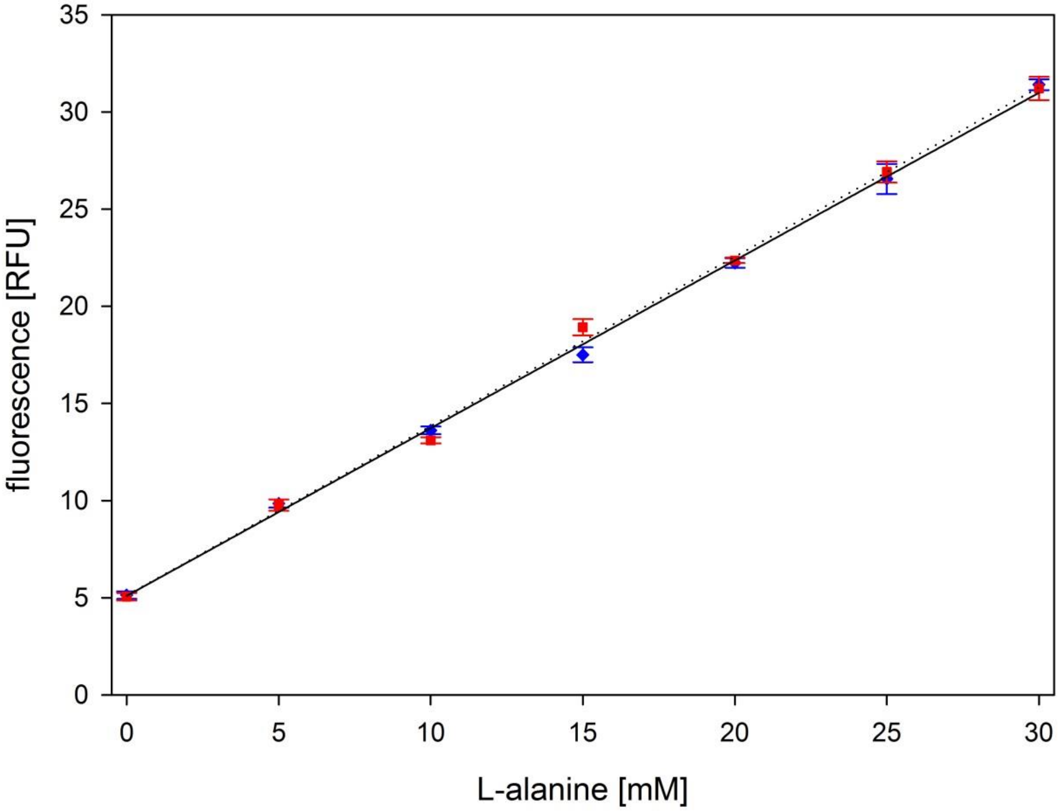

Figure S1. Fluorescamine calibration curves in different buffer matrices. Ammonium is consumed during the reductive amination of pyruvate to L-alanine. To exclude effects of reduced ammonium concentration on the quantification of L-alanine, we compared standards containing either 200 mM ammonium or an ammonium concentration that is reduced by the concentration of L-alanine in the corresponding standard sample. Blue diamonds: 100 mM HEPES pH 7.35, 200 mM ammonium; red squares: 100 mM HEPES pH 7.35, (200 mM – [L-alanine]) ammonium. Fluorescamine assays were performed with 100-fold diluted samples in total reaction volumes of 158 µL, average values of technical triplicates are shown with error bars indicating the standard deviation.

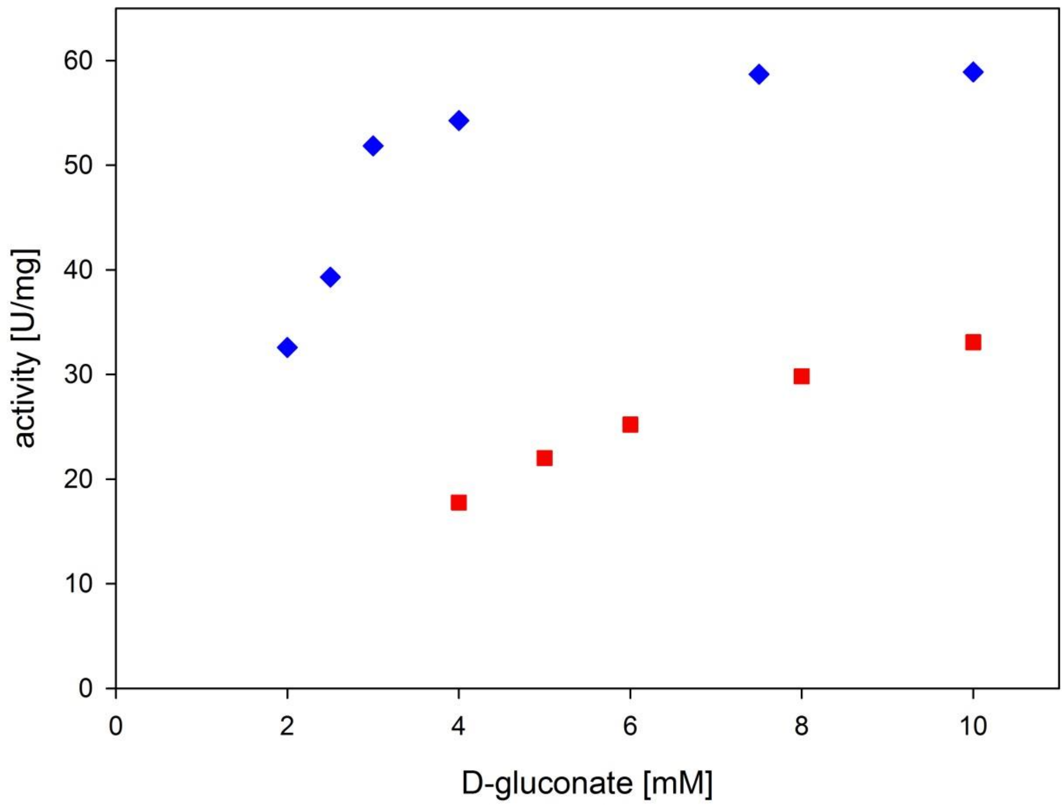

Figure S2. Kinetic measurements of CcDHAD containing 2.2 mM glycerate (red squares) compared to standard conditions (blue diamonds). Reactions were performed at 50 °C in 100 mM HEPES buffer pH 7.35 and stopped after 10 min by enzyme removal. Activities were calculated from the residual amount of D-gluconate detected by HPLC.

Expressed DNA Sequences

Bold nucleotides represent additional tags from the expression plasmid.

BsGDH (NCBI-Prot. ID: NP_388275.1)

ATGTATCCGGATTTAAAAGGAAAAGTCGTCGCTATTACAGGAGCTGCTTCAGGGCTCGGAAAGGCGATGGCCATTCGCTTCGGCAAGGAGCAGGCAAAAGTGGTTATCAACTATTATAGTAATAAACAAGATCCGAACGAGGTAAAAGAAGAGGTCATCAAGGCGGGCGGTGAAGCTGTTGTCGTCCAAGGAGATGTCACGAAAGAGGAAGATGTAAAAAATATCGTGCAAACGGCAATTAAGGAGTTCGGCACACTCGATATTATGATTAATAATGCCGGTCTTGAAAATCCTGTGCCATCTCACGAAATGCCGCTCAAGGATTGGGATAAAGTCATCGGCACGAACTTAACGGGTGCCTTTTTAGGAAGCCGTGAAGCGATTAAATATTTCGTAGAAAACGATATCAAGGGAAATGTCATTAACATGTCCAGTGTGCACGAAGTGATTCCTTGGCCGTTATTTGTCCACTATGCGGCAAGTAAAGGCGGGATAAAGCTGATGACAAAGACATTAGCGTTGGAATACGCGCCGAAGGGCATTCGCGTCAATAATATTGGGCCAGGTGCGATCAACACGCCAATCAATGCTGAAAAATTCGCTGACCCTAAACAGAAAGCTGATGTAGAAAGCATGATTCCAATGGGATATATCGGCGAACCGGAGGAGAT

CGCCGCAGTAGCAGCCTGGCTTGCTTCGAAGGAAGCCAGCTACGTCACAGGCATCACGTTATTCGCGGACGGCGGTATGACACTCTATCCTTCATTCCAGGCAGGCCGCGGTTAA

CcDHAD (NCBI-Prot. ID: WP_012640069.1)

ATGGGCAGCAGCCATCATCATCATCATCACAGCAGCGGCCTGGTGCCGCGCGGCAGCCATATGAGCAATCGTACACCGCGTCGTTTTCGTAGCCGTGATTGGTTTGATAATCCGGATCATATTGATATGACCGCACTGTATCTGGAACGCTTTATGAATTATGGTATCACACCGGAAGAACTGCGTAGCGGTAAACCGATTATTGGTATTGCACAGACCGGTAGCGATATTAGCCCGTGTAATCGTATTCATCTGGATCTGGTTCAGCGTGTTCGTGATGGTATTCGTGATGCCGGTGGTATTCCGATGGAATTTCCGGTTCATCCGATTTTTGAAAATTGTCGTCGTCCGACCGCAGCACTGGATCGTAATCTGAGCTATCTGGGTTTAGTTGAAACCCTGCATGGTTATCCGATTGATGCAGTTGTTCTGACCACCGGTTGTGATAAAACCACACCGGCAGGTATTATGGCAGCAACCACCGTTAATATTCCGGCAATTGTTCTGAGCGGTGGTCCGATGCTGGATGGTTGGCATGAAAATGAACTGGTTGGTAGCGGCACCGTTATTTGGCGTAGCCGTCGTAAACTGGCAGCCGGTGAAATTACCGAAGAGGAATTTATTGATCGTGCAGCAAGCAGCGCACCGAGCGCAGGTCATTGCAATACAATGGGCACCGCAAGCACCATGAATGCAGTTGCAGAAGCACTGGGTCTGAGCCTGACCGGTTGCGCAGCAATTCCGGCACCGTATCGTGAACGTGGTCAGATGGCATATAAAACAGGTCAGCGTATTGTTGATCTGGCCTATGATGATGTTAAACCGCTGGATATTCTGACCAAACAGGCATTTGAAAATGCAATTGCCCTGGTTGCAGCAGCAGGCGGTAGCACCAATGCACAGCCGCATATTGTTGCAATGGCACGTCATGCCGGTGTTGAAATTACAGCAGATGATTGGCGTGCAGCCTATGATATTCCGCTGATTGTTAATATGCAGCCTGCAGGTAAATATCTGGGTGAACGTTTTCATCGTGCCGGTGGCGCTCCGGCAGTTCTGTGGGAACTGTTACAGCAGGGTCGTCTGCATGGTGATGTGCTGACCGTTACCGGCAAAACCATGAGCGAAAATCTGCAGGGTCGCGAAACCAGCGATCGTGAAGTTATTTTTCCGTATCATGAACCGCTGGCAGAAAAAGCAGGTTTTCTGGTTCTGAAAGGTAACCTGTTTGATTTTGCCATCATGAAAAGCAGCGTTATCGGTGAAGAATTTCGTAAACGTTATCTGAGCCAGCCTGGTCAAGAAGGTGTTTTTGAAGCACGTGCAATTGTGTTTGATGGTAGCGACGATTATCATAAGCGTATTAATGATCCGGCACTGGAAATTGATGAACGTTGCATTCTGGTTATTCGTGGTGCAGGTCCGATTGGTTGGCCTGGTAGTGCCGAAGTTGTGAACATGCAGCCACCGGACCATCTGCTGAAAAAAGGCATTATGAGCCTGCCGACCTTAGGTGATGGTCGTCAGAGTGGCACCGCAGATAGCCCGAGCATTCTGAATGCAAGTCCGGAAAGCGCAATTGGTGGTGGTCTGAGCTGGCTGCGTACCGGTGATACAATTCGTATTGATCTGAATACCGGTCGTTGTGATGCACTGGTTGATGAAGCAACCATTGCAGCACGTAAACAGGATGGCATTCCTGCAGTTCCGGCAACCATGACACCGTGGCAAGAAATTTATCGTGCACATGCAAGCCAGCTGGATACCGGTGGTGTTCTGGAATTTGCCGTTAAATATCAGGATCTGGCAGCAAAACTGCCTCGCCATAATCATTAA

PtKDGA (NCBI-Prot. ID: 4UXD_A)

ATGGGCAGCAGCCATCATCATCATCATCACAGCAGCGGCCTGGTGCCGCGCGGCAGCCATATGTACAAGGGAATCGTTTGTCCGATGATTACCCCGCTGGACGCACACGGCAACATCGACTATAACGCAACGAACATTCTGATCAAATACCTGGAAGGCATCAACGTGGACTACCTGTTCCCGATGGGCTCTACCGGTGTGTTTCCGTATTTCACGCTGAAAGAACGTAAAGACTTTCTGAAATTCGTTCGCGAAAACAGTAAAAAACCGATCATGGCTGGCGTCGGTAGCTCTAGTATCAACGAAGTGAACGAACTGATGAAATTCAGCATGGATATCGGTATCGAAGCGGCCGTGCTGATGCCGCCATATTACATTAAACTGAACCAGGAAGCAATCTACCATTACTACAAAGAAATCCTGTCCTCAAATGACATGGATCTGCTGATTTACAACATCCCGCAATTTACCAATAAAATCGATCCGGAAA

CGGTCAAAAACCTGAAATCTGAATTTAGCAGCGTGAAAGGTGTTAAAGACTCTAGTGCAGAT
ATTCGTGGCTTTATGGAAATGCTGTCCCTGTCAGATGACGATTTTGCTGTTTTCCAGGGTCA
AGACGATCTGCTGTTTACCTCGCTGGAACTGGGTGCCAGCGGCGGTGTTTGTGGTACCAC
GAATTTTAGCGACGGCATCGTCCGTCTGTATCACGAATACAAAAACAATCGCGAAATGGCC
CTGAAAATTGAGAAAAACGATGTTATCCCGCTGATGAAAAAACTGGGCAAATATCAGTTTCC
GAATGCATATTACGAATATTTCTACAAGAAAAACAATATCAATGGCGGCTACCGTCCGCCGA
TGTATCGCGTTGGCATCGAAATCTAA

MjALDH (NCBI-Prot. ID: WP_010870928)

ATGTTTATTGATGGCAAATGGATTAACCGCGAGGATATGGATGTTATTAATCCGTATAGCCT
GGAAGTGATCAAAAAAATCCCTGCACTGAGCCGTGAAGAAGCAAAAGAAGCCATTGATACC
GCAGAGAAATACAAAGAAGTCATGAAAAATCTGCCGATCACCAAACGCTATAACATCCTGAT
GAATATTGCCAAGCAGATCAAAGAAAAAAAAGAAGAACTGGCCAAAATCCTGGCAATTGAT
GCAGGTAAACCGATTAAACAGGCACGTGTTGAAGTTGAACGTAGCATTGGCACCTTTAAAC
TGGCAGCCTTTTATGTGAAAGAACACCGTGATGAAGTTATTCCGAGTGATGATCGTCTGATT
TTTACCCGTCGTGAACCGGTTGGTATTGTTGGTGCAATTACCCCGTTTAACTTTCCGCTGAA
TCTGAGCGCACATAAAATTGCACCGGCAATTGCAACCGGTAATGTTATTGTTCATCATCCGA
GCAGCAAAGCACCGCTGGTTTGTATTGAACTGGCAAAGATTATTGAGAACGCCCTGAAAAA
ATACAATGTTCCGCTGGGTGTTTATAATCTGCTGACCGGTGCCGGTGAAGTTGTTGGTGAT
GAAATTGTTGTGAACGAGAAGGTGAACATGATTAGCTTTACCGGTAGCAGTAAAGTGGGCG
AACTGATTACCAAAAAAGCCGGTTTTAAGAAAATCGCCCTGGAATTAGGTGGTGTGAATCC
GAATATTGTTCTGAAAGATGCCGATCTGAATAAAGCAGTTAACGCGCTGATTAAAGGCAGCT
TTATCTATGCAGGTCAGGTTTGCATTAGCGTTGGTATGATTCTGGTTGATGAAAGCATTGCC
GATAAGTTCATCGAAATGTTTGTGAACAAAGCCAAAGTGCTGAATGTTGGTAATCCGCTGGA
TGAAAAAACCGATGTTGGTCCGCTGATTAGCGTGGAACATGCAGAATGGGTTGAAAAAGTT
GTGGAAAAAGCGATTGATGAAGGTGGCAAACTGCTGTTAGGTGGTAAACGTGATAAAGCAC
TGTTTTATCCGACCATTCTGGAAGTGGATCGTGATAATATTCTGTGCAAAACCGAAACCTTT
GCACCGGTGATTCCGATTATTCGTACCAATGAAGAAGAAATGATCGATATTGCGAACAGCA
CCGAATATGGTCTGCATAGCGCAATTTTTACCAACGACATTAACAAGAGCCTGAAATTTGCC
GAAAATCTGGAATTTGGCGGTGTTGTGATTAATGATAGCAGCCTGTTTCGTCAGGATAACAT
GCCGTTTGGTGGCGTGAAAAAAAGCGGTCTGGGTCGTGAAGGTGTTAAATATGCAATGGAA
GAGATGAGCAACATCAAAACCATCATCATCAGCAAATAA

AfAlaDH (NCBI-Prot. ID: WP_010879161.1)

ATGGAAACCCTGATCCTGACGCAAGAAGAAGTTGAAAGCCTGATTAGCATGGATGAAGCAA
TGAATGCAGTTGAAGAGGCATTTCGTCTGTATGCACTGGGTAAAGCACAGATGCCTCCGAA
AGTTTATCTGGAATTTGAAAAAGGTGATCTGCGTGCAATGCCTGCACATCTGATGGGTTATG
CAGGTCTGAAATGGGTTAATAGCCATCCGGGTAATCCGGATAAAGGTCTGCCGACCGTTAT
GGCACTGATGATTCTGAATAGTCCGGAAACAGGTTTTCCGCTGGCAGTTATGGATGCAACC
TATACCACCAGTCTGCGTACCGGTGCAGCCGGTGGTATTGCAGCAAAATATCTGGCACGTA
AAAATAGCAGCGTGTTTGGTTTTATTGGTTGTGGCACCCAGGCATATTTTCAGCTGGAAGCA
CTGCGTCGTGTTTTTGATATTGGTGAAGTTAAAGCGTATGACGTGCGTGAAAAAGCAGCCA
AAAAATTCGTTAGCTATTGCGAAGATCGTGGTATTAGCGCAAGCGTTCAGCCTGCCGAAGA
GGCAAGCCGTTGTGATGTTCTGGTTACCACCACACCGAGCCGTAAACCGGTTGTTAAAGCA
GAATGGGTAGAAGAGGGCACCCATATTAATGCAATTGGTGCAGATGGTCCGGGTAAACAAG

AACTGGATGTTGAAATTCTGAAGAAAGCCAAAATCGTGGTGGATGATCTGGAACAGGCAAAACATGGTGGTGAAATTAATGTTGCAGTTAGCAAAGGTGTGATTGGCGTTGAAGATGTTCATGCAACCATTGGCGAAGTTATTGCTGGCCTGAAAGATGGTCGTGAAAGTGATGAAGAAATCACCATTTTTGATAGCACCGGTCTGGCAATTCAGGATGTTGCCGTTGCAAAAGTTGTTTATGAAAATGCCCTGAGCAAAAACGTGGGTAGCAAAATCAAATTTTTCCGCATC

SsDHAD (NCBI-Prot. ID: WP_012953192.1)

ATGCCTGCAAAACTGAATAGCCCGAGCCGTTATCATGGTATTTATAATGCACCGCATCGTGCATTTCTGCGTAGCGTTGGTCTGACCGATGAAGAAATTGGTAAACCGCTGGTTGCAATTGCCACCGCATGGTCTGAAGCCGGTCCGTGTAATTTTCATACCCTGGCACTGGCACGTGTTGCAAAAGAAGGCACCAAAGAAGCCGGTCTGTCTCCGCTGGCATTTCCGACCATGGTTGTGAATGATAATATTGGCATGGGTAGCGAAGGTATGCGTTATAGCCTGGTTAGCCGTGATCTGATTGCAGATATGGTTGAAGCACAGTTTAATGCCCATGCATTTGATGGTCTGGTTGGTATTGGTGGTTGTGATAAAACCACACCGGGTATTCTGATGGCAATGGCACGTCTGAATGTTCCGAGCATTTATATTTATGGTGGTAGCGCAGAACCGGGTTATTTTATGGGTAAACGCCTGACCATTGAAGATGTTCATGAAGCCATTGGTGCATATCTGGCAAAACGCATTACCGAAAATGAACTGTATGAAATTGAAAAACGTGCACATCCGACCCTGGGCACCTGTAGCGGTCTGTTTACCGCAAATACCATGGGTAGCATGAGCGAAGCACTGGGTATGGCACTGCCTGGTAGCGCATCTCCGACCGCAACCAGCAGCCGTCGTGTTATGTATGTTAAAGAAACCGGTAAAGCCCTGGGTAGCCTGATTGAAAATGGCATTAAAAGCCGTGAAATTCTGACCTTTGAAGCCTTTGAAAATGCAATTACAACCCTGATGGCGATGGGTGGTAGCACCAATGCAGTTCTGCATCTGCTGGCAATTGCTTATGAAGCCGGTGTTAAACTGACCCTGGATGATTTTAATCGCATTAGCAAACGCACCCCGTATATTGCAAGCATGAAACCGGGTGGTGATTATGTTATGGCCGATCTGGATGAAGTTGGTGGTGTTCCGGTTGTTCTGAAAAAACTGCTGGATGCCGGTCTGCTGCATGGTGATGTTCTGACCGTTACCGGTAAAACCATGAAACAGAATCTGGAACAGTATAAATATCCGAATGTGCCGCATAGCCATATTGTTCGTGATGTGAAAAATCCGATTAAACCGCGTGGTGGTATTGTTATTCTGAAAGGTAGCCTGGCACCGGAAGGTGCAGTTATTAAAGTTGCAGCCACCAATGTGGTTAAATTTGAAGGCAAAGCCAAAGTGTATAATAGCGAAGATGATGCCTTTAAAGGTGTTCAGAGCGGTGAAGTTAGCGAAGGTGAAGTGGTGATTATTCGCTATGAAGGTCCGAAAGGTGCACCGGGTATGCCGGAAATGCTGCGCGTTACCGCAGCGATTATGGGTGCCGGTCTGAATAATGTTGCACTGGTTACCGATGGTCGTTTTAGCGGTGCAACCCGTGGTCCGATGGTTGGTCATGTTGCACCGGAAGCAATGGTTGGTGGTCCGATTGCAATTGTTGAAGATGGCGATACCATTGTGATTGATGTGGAAAGCGAACGTCTGGATCTGAAACTGAGCGAAGAAGAAATTAAAAATCGCCTGAAACGTTGGAGCCCGCCGTCACCGCGTTATAAAAGCGGTCTGCTGGCAAAATATGCAAGCCTGGTTTCTCAGGCAAGCATGGGTGCAGTTACCCGTCCGGCATAA
